# Case Report: A novel method of needle-free transseptal puncture

**DOI:** 10.3389/fcvm.2024.1493240

**Published:** 2024-12-03

**Authors:** Chia-Chen Lee, Chen-An Chao, Su-Huan Chang, Chun-Kai Chen, Yen-Siou Chen, Chang-En Lin, Tsung-Ping Jeng, Chih-Chieh Yu

**Affiliations:** ^1^ Department of Internal Medicine, College of Medicine, National Taiwan University, Taipei, Taiwan; ^2^ Division of Cardiology, Department of Internal Medicine, National Taiwan University College of Medicine and Hospital, Taipei, Taiwan; ^3^ Department of Cardiology, Fu Jen Catholic University Hospital, Taipei, Taiwan

**Keywords:** electrophysiology, atrial fibrillation, transseptal puncture, intracardiac echocardiography, electrocautery

## Abstract

**Background:**

In the era of fluoroless catheter ablation (CA), achieving a successful transseptal puncture (TSP) presents a significant challenge. We introduce a novel technique for zero-fluoroscopy and cost-effective needle-free TSP.

**Case summary:**

We describe two cases where a GMS-1 guidewire (0.025 inch, pigtail configuration; Toray Medical Co., Ltd., Japan) was utilized for TSP. This technique was performed using either fluoroscopy or intracardiac echocardiography (ICE). The procedure was completed successfully in both cases, with no complications reported.

**Conclusion:**

The use of a 0.025 inch GMS-1 guidewire with an electrocautery technique enables effective transseptal puncture without the need for a needle or fluoroscopy. This novel approach offers a safe, efficient, and zero-fluoroscopic alternative for TSP.

## Introduction

A successful transseptal puncture (TSP) is essential to gain access to the left atrium (LA) during catheter ablation procedures for atrial fibrillation (AF) (1). TSP has become the standard approach to cross the atrial septum safely. Traditionally, TSP is performed using an SL0 sheath in combination with a Brockenbrough (BRK) transseptal needle (St. Jude Medical, USA) (2). After crossing the septum, operators often exchange the initial 0.032 inch J-tip guidewire for a GMS-1 guidewire (0.025 inch, pigtail configuration; Toray Medical Co., Ltd., Japan) to facilitate device advancement into the LA.

Since the first TSP procedure in the late 1950s (3–6), numerous technical advancements have been integrated into clinical practice (7–11), including the adoption of zero-fluoroscopic TSP under intracardiac echocardiography (ICE) guidance (12). However, in our practice, we identified several drawbacks associated with performing fluoroless TSP using either the BRK needle or electrocautery guidewire. The BRK needle often falls out of the echocardiographic frame, increasing the risk of atrial wall perforation due to poor visualization of the needle tip. Additionally, the J-tip guidewire of the SL0 sheath often provides suboptimal support, complicating septal crossing with the SL0 sheath.

**Figure 1:**
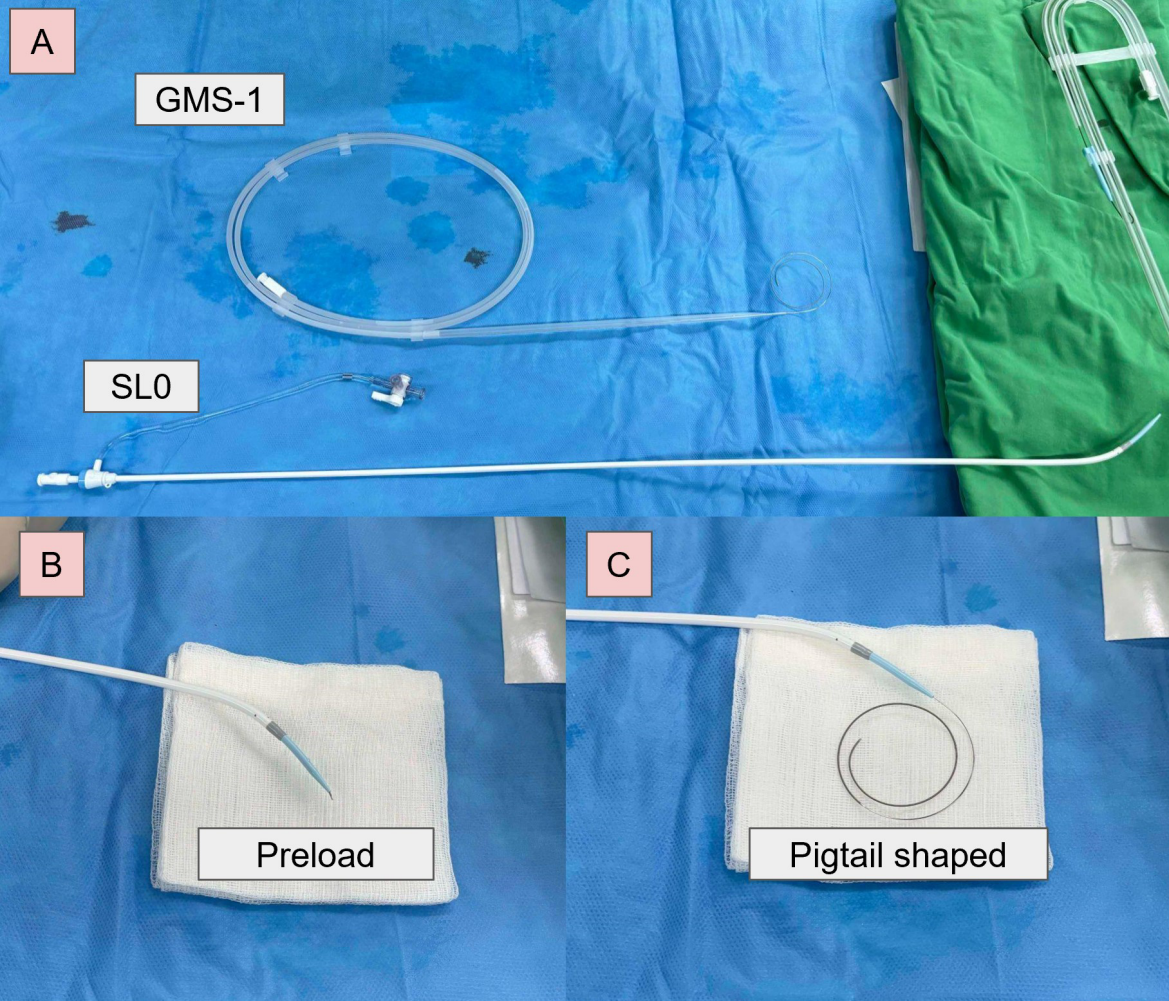
GMS-1 guidewire-based electrocautery TSP. **(A)** SL0 sheath and a GMS-1 guidewire. **(B)** Preloaded before TSP. **(C)** The tip of the guidewire formed a pigtail configuration after crossing.

In response to these challenges, we explored the feasibility of performing TSP directly using a 0.025 inch GMS-1 guidewire (Figure 1). This approach may offer a more efficient and safer alternative to traditional methods.

## Case report

### Case 1

A 61-year-old male was referred to our hospital for radiofrequency pulmonary vein isolation (PVI) ablation due to highly symptomatic, drug-refractory paroxysmal atrial fibrillation (AF). Additionally, a computed tomography (CT) scan was performed to merge morphological and electroanatomic information during the ablation.

The procedure was performed under intravenous general anesthesia. An intracardiac echocardiography (ICE) catheter (SoundStar, Biosense Webster) was introduced via the left femoral vein, providing optimal imaging of the atrium and interatrial septum. After creating an anatomical map with real-time ICE and CARTO-Sound Image Integration Module, a merged CT and Sound map was acquired. We placed a 10-pole catheter into the coronary sinus under CARTO and ICE guidance. Subsequently, a 0.032 inch guidewire was advanced from the right femoral vein to the superior vena cava (SVC) to guide the transseptal sheath (SL0, St. Jude Medical). Once the sheath reached the SVC, the 0.032 inch guidewire was exchanged for a GMS-1 guidewire (0.025 inch, pigtail-shaped). Under real-time visualization, the transseptal sheath (SL0, St. Jude Medical) was introduced into the right atrium (RA).

**Figure 2:**
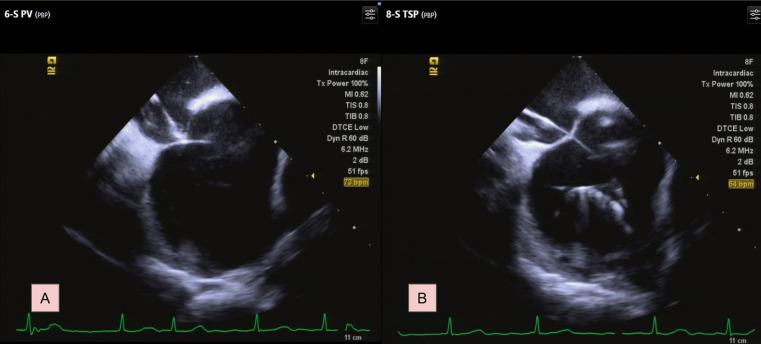
Transseptal process under ICE. **(A)** Identify the septum by typical tenting image. **(B)** The GMS-1 guidewire formed a pigtail shaped. For a complete video clip, please refer to Supplementary Video S1.

Under ICE guidance, the sheath and the GMS-1 wire were positioned against the septum. This was confirmed by the typical “tenting” appearance on echocardiographic imaging when slight pressure was applied to the septal wall (Figure 2A). The wire was then advanced through the septum into the left atrium (LA) using electrocautery technique with an energy of 30–35 W for less than 1 s under “dry cut” mode by ERBE VIO 10°C generator (ERBE Elektromedizin GmbH, Germany). Upon accessing the LA, the wire's tip immediately assumed a pigtail configuration, preventing further tissue penetration (Figure 2B; Supplementary Video S1). Subsequently, the SL0 sheath and its dilator were smoothly introduced into the LA. A second TSP with Vizigo (Biosense Webster) was done smoothly using the same technique. PVI was subsequently completed smoothly.

### Case 2

A 69-year-old female presented to our center for radiofrequency ablation with PVI due to symptomatic persistent AF. Pre-procedural TEE confirmed the absence of thrombi in the LA appendage. As in the previous case, both TEE and a CT scan were conducted pre-procedurally.

The procedure was conducted under intravenous general anesthesia. A 10-pole catheter was first introduced into the coronary sinus. Subsequently, a 0.032 inch guidewire was advanced from the right femoral vein to the SVC to guide the transseptal sheath (SL0, St. Jude Medical). Once the sheath reached the superior vena cava (SVC), the 0.032 inch guidewire was exchanged for a GMS-1 guidewire (0.025 inch, pigtail-shaped).

**Figure 3:**
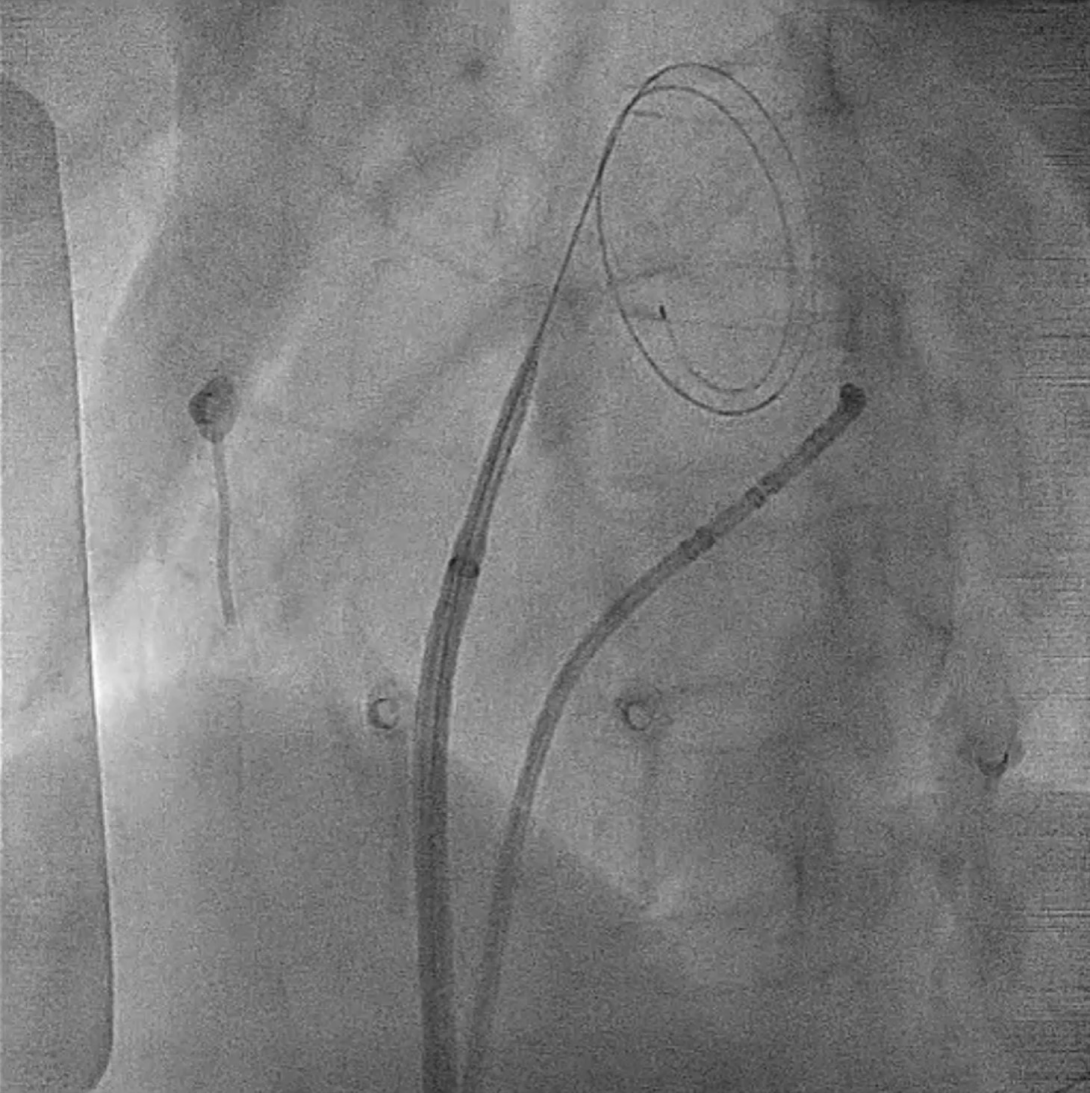
Transseptal process under fluoroscopy. The GMS-1 guidewire became pigtail configuration after crossing the septum. For a complete video clip, please refer to Supplementary Video S2.

Under fluoroscopic guidance, the interatrial septum was identified, and the tip of the guidewire was positioned against the septum. TSP was then successfully performed using the electrocautery technique similar as above (Figure 3; Supplementary Video S2), allowing the smooth introduction of the SL0 sheath and its dilator into the LA. A second puncture with Agilis NxT Steerable Introducer (Abbott Medical Australia Pty Ltd) was performed using the same technique. The catheter ablation was subsequently completed without complications.

## Discussion

In this case report, we demonstrate a novel electrocautery-assisted TSP technique using the cost-effective and widely accessible GMS-1 guidewire. This approach not only enhances the safety of TSP by providing additional support when advancing the sheath and reducing the need for guidewire exchanges within the LA, but also improves procedural efficiency. This technique is particularly advantageous when employing two-dimensional ICE to pursue a zero-fluoroscopy procedure.

Electrocautery-assisted TSP using guidewires has become increasingly utilized among experienced operators in modern era (7, 8, 10, 12, 13). Compared with previous studies, our approach offers several key advantages:

1. **Safety**: Unlike TSP using traditional mechanical needles, the GMS-1 guidewire immediately assumes a pigtail configuration upon crossing the septum, which minimizes the risk of atrial wall perforation (7, 8). This is particularly beneficial during second TSP with ICE guidance, because the image under ICE was often interfered by the first transseptal sheath.
2. **Efficiency**: The absence of a need for guidewire exchange streamlines the procedure, reducing the time required and mitigating the risk of thrombus formation or air bubble introduction.
3. **Support for Large Sheath Exchange**: The GMS-1 guidewire is designed to facilitate the smooth delivery of large sheaths, such as the SL0 sheath or steerable sheath, through the septum, enhancing procedural efficiency and ease of use.
4. **Cost-effective**: Although several commercial kits have demonstrated the efficacy and safety in needle-free and fluoroless TSP, their cost still remains a significant concern (7, 11). Our technique offers a more affordable alternative with comparable efficacy.

With the rising popularity of single-shot procedures, including cryoablation and pulsed field ablation, larger sheaths are increasingly required (14–16). In our experience, we have successfully delivered a cryoablation sheath (Medtronic, FlexCath Advance™ steerable sheath, 15 Fr) using the GMS-1 guidewire, suggesting that our technique could support these larger sheaths effectively. Further research and case studies are warranted to evaluate the technique's broader applicability in this context.

In conclusion, we presented two cases demonstrating the safety, efficacy, and cost-effectiveness of needle-free and zero-fluoroscopic electrosurgery-assisted TSP using a 0.025 inch GMS-1 guidewire. This approach appears to be a viable and economical alternative to traditional methods.

## Data Availability

The original contributions presented in the study are included in the article/Supplementary Material, further inquiries can be directed to the corresponding author.

## Supplementary material

Supplementary Video S1 A video clip showed the moment of atrial septal crossing under fluoroscopy. The guidewire curved immediately after a successful TSP.

Supplementary Video S2 A video clip showed the moment of atrial septal crossing under ICE. TSP, transseptal puncture; ICE, intracardiac echocardiography.
